# Case report: Perinephric lymphangiomatosis

**DOI:** 10.4103/0971-3026.69364

**Published:** 2010-08

**Authors:** Rajani Gorantla, Anusheela Yalapati, Bhawna Dev, Santhosh Joseph

**Affiliations:** Department of Radiology and Imaging Sciences, Sri Ramachandra Medical College and Research Institute, Sri Ramachandra University, Chennai - 600 116, India

**Keywords:** Perirenal lymphangiomatosis, perirenal collections, kidney

## Abstract

Perirenal lymphangiomatosis is a rare benign malformation of the lymphatic system. We report here a case of bilateral perirenal and parapelvic involvement with a normal excretory collecting system.

## Introduction

Renal lymphangiomatosis is a rare benign disorder of renal lymphatics, which is often confused with other cystic diseases of the kidney.[1] Definitive diagnosis is possible with percutaneous needle aspiration of chylous fluid.[2] However, the USG and CT scan findings are also quite characteristic and allow easy diagnosis.[3]

## Case Report

A 15-year-old girl presented with a history of vague abdominal pain and loss of appetite. There was no significant past or family history of any diseases. On examination, the blood pressure was 140/60 mm Hg. Laboratory analysis revealed normal renal function tests. Urinary examination was normal. Blood investigations were unremarkable.

**Figure 1 (A,B):**
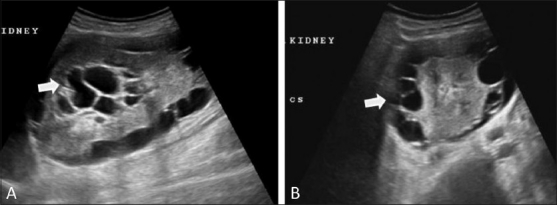
Longitudinal (A) and transverse (B) USG images show perirenal collections (arrows) with septae

USG showed multiloculated, almost symmetrical, bilateral perirenal collections [Figure 1A and B] with septae and internal echoes. The collections were also seen to insinuate into the renal sinuses. Both kidneys were normal in size and the corticomedullary differentiation was maintained.

**Figure 2:**
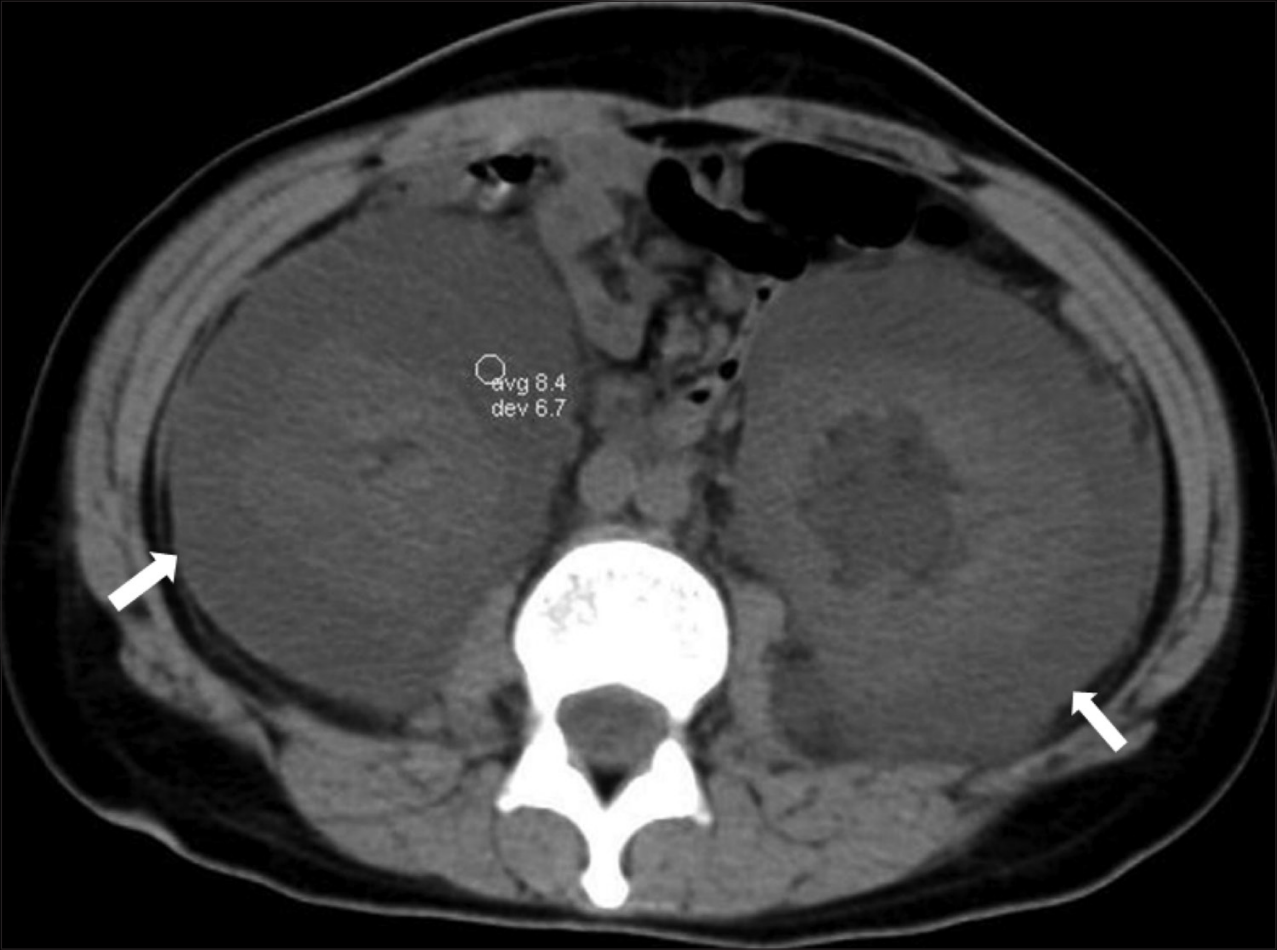
Non-enhanced, axial CT scan shows symmetric, hypodense perirenal collections (arrows) with an average Hounsfield value of 8

**Figure 3 (A,B):**
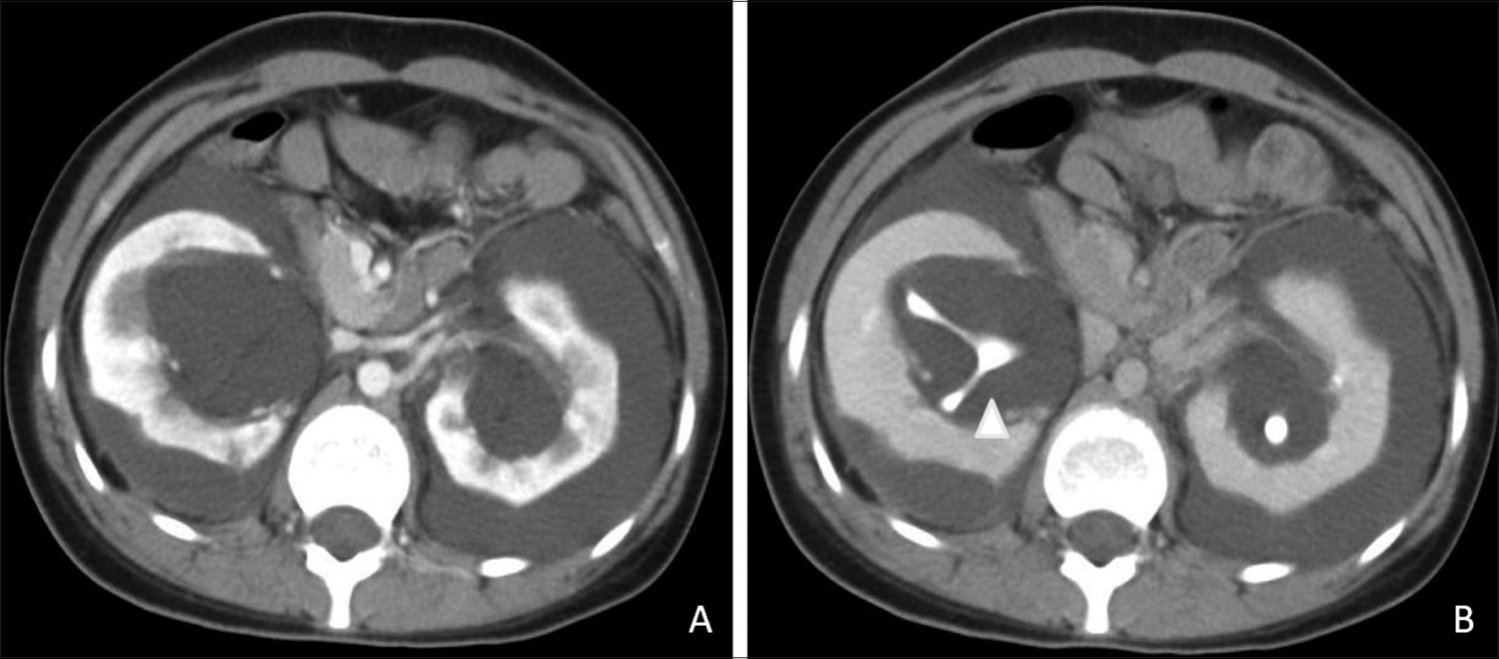
Contrast-enhanced CT scans in the corticomedullary (A) and excretory (B) phases show normal cortical enhancement with nonenhancing perirenal collections. The excretory images show splaying of the non-dilated excreting collecting system (arrowhead)

CT scan of the abdomen showed bilateral hypodense collections [Figure 2] with densities varying between 5 and 15 HU in the perirenal and peripelvic regions. Normal enhancement of the cortex [Figure 3A] and normal corticomedullary differentiation were seen. The collections were seen indenting the cortices of both kidneys. The inferior venacava was displaced anteromedially.

**Figure 4:**
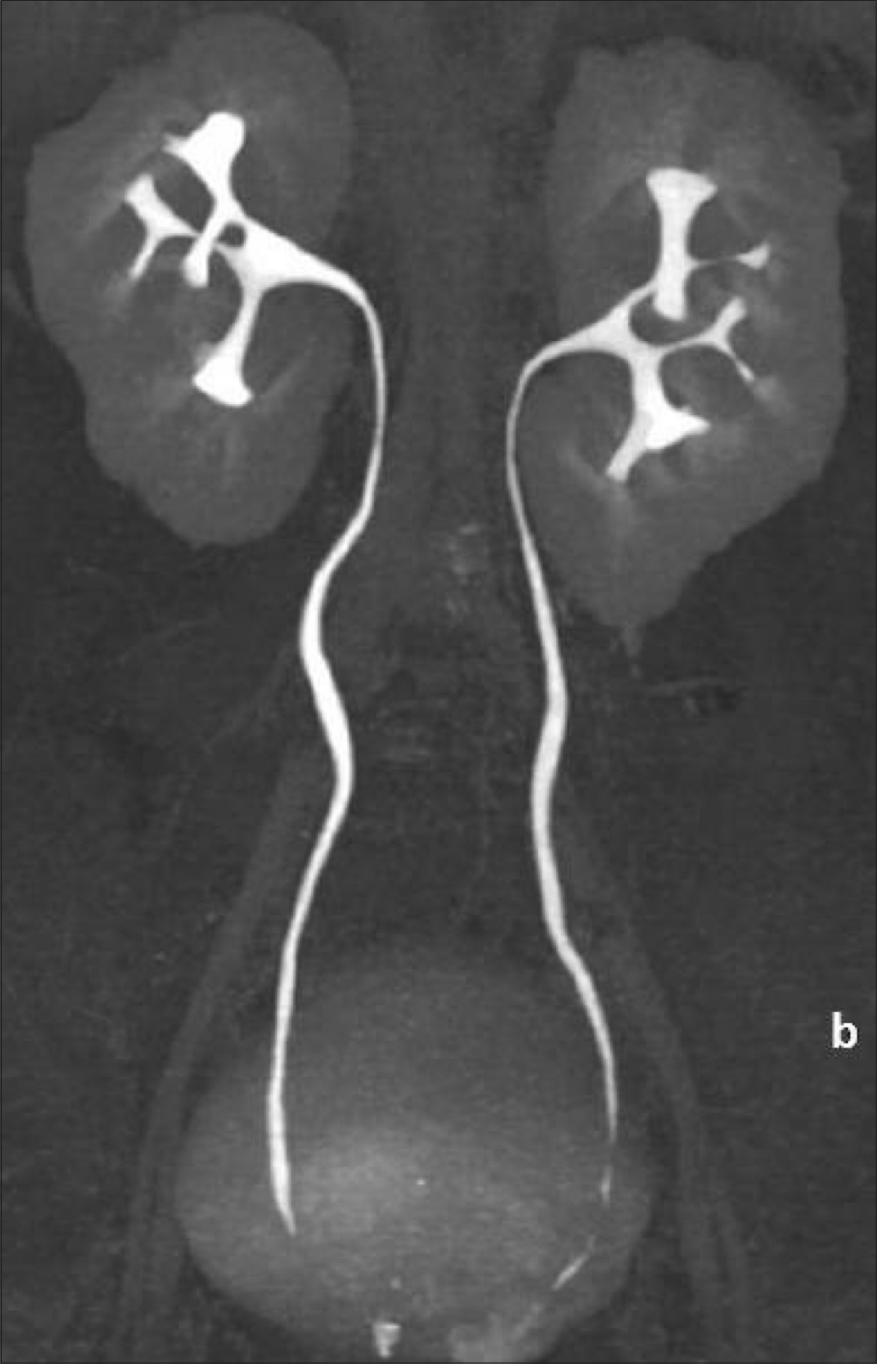
CT urogram. A maximum intensity projection, coronal CT scan image shows splayed and non-dilated excreting collecting systems

Delayed scans obtained after 15 min [Figure 3B] showed normal excretion, but splayed pelvi-calyceal systems [Figure 4] due to the intervening fluid within the sinuses. No invasion of the pelvi-calyceal systems was noted and no extravasation of contrast into the perirenal collections was seen.

These findings were considered suggestive of of perirenal lymphangiomatosis.

USG-guided aspiration of the perirenal fluid was performed as part of the treatment. The fluid was sent for biochemical analysis and it revealed a few lymphocytes with abundant proteins and renins specific to the kidneys. No organisms were isolated from the aspirated fluid.

## Discussion

Renal lymphangiomatosis or lymphangiectasis is a rare, benign disorder, characterized by dilated perirenal, intrarenal and parapelvic lymphatic structures. This is due to a failure of renal lymphatic drainage into the retroperitoneal lymphatics, subsequently causing dilatation of the ducts and formation of unilocular or multifocal cystic spaces in the perirenal and renal sinus regions. They can occur at any age group. This condition may be asymptomatic or may present with flank pain, hypertension, proteinuria and hematuria.[4]

USG shows multiloculated, cystic perirenal and parapelvic collections with thin septae. The kidneys may be normal or enlarged in size. The differential diagnosis is usually with polycystic kidneys and hydronephrosis. In polycystic kidneys, multiple cysts are seen within the cortex, whereas the lesions in lymphangiomatosis are in the perirenal and pararenal locations with normal renal parenchyma.[1] In hydronephrosis, the pelvi-calyceal system is dilated, whereas in lymphangiomatosis, splaying of the calyces is seen. In infants with perirenal lymphangiomatosis, USG shows hyperechoic kidneys that need to be differentiated from infantile polycystic kidneys due to similar imaging appearances, though the renal function is normal in lymphangiomatosis.[5]

CT scans show well-defined hypodense collections in the perirenal and parapelvic spaces with septae. The renal parenchyma is normal.[2] No abnormal enhancement is seen within the collections. CT urogram images show splaying of the renal calyceal system with normal function. On CT scan, the presence of fluid attenuation in the range of 0–10 HU with absence of enhancement excludes other entities such as nephroblastomatosis and lymphoma. The other differential diagnoses of perirenal fluid collections are urinoma, hematoma and abscess. All these can be differentiated from lymphangiomatosis on the basis of whether the disease is bilateral or unilateral, the condition of the underlying parenchyma and the attenuation and enhancement patterns of the collections.[6]

MRI shows multiple hyperintense collections with septae on T2W images with reversal of the corticomedullary intensity,[7] which is due to an anatomic variation in the lymphatics, with abundance around the interlobar and arcuate blood vessels at the cortico medullary junction, fewer small lymphatics in the mid-cortex region and absence of lymphatics in the medulla region.[8]

The diagnosis is made by aspiration of chyle rich in lymphocytes.[4] The presence of renin is more specific and confirms the renal origin of the collections.
